# Tale of a Wandering Lead: Late Atrial Lead Perforation into Right Lung following Pacemaker Implantation

**DOI:** 10.7759/cureus.1865

**Published:** 2017-11-21

**Authors:** Arjun Saradna, Ankur Sinha, Madina Abduraimova, Daniel Rodriguez, Felix Yang

**Affiliations:** 1 Internal Medicine, Maimonides Medical Center; 2 Cardiology, Maimonides Medical Center; 3 Electrophysiology, Maimonides Medical Center

**Keywords:** pacemaker, perforation, lung, displaced, penetrating

## Abstract

Cardiac perforation by a pacemaker lead is a rare complication of pacemaker implantation. Presentation can vary from chest pain and shortness of breath to the patient being completely asymptomatic. Diagnosis is usually made by high-resolution computed tomography (HRCT) scan of the chest. Electrocardiograph (EKG) usually shows the absence of a paced rhythm, but it doesn't provide a definitive diagnosis. We describe a case of late cardiac perforation by an atrial pacemaker lead with no signs or symptoms of pericardial tamponade.

## Introduction

Cardiac perforation by a pacemaker lead is a rare complication of pacemaker implantation [1]. Symptoms can range from mild pain to severe life-threatening conditions like cardiac tamponade. We describe a rare case of late right atrial, pericardial, and right lung parenchymal perforation by an atrial pacemaker lead with no signs or symptoms of pericardial tamponade.

## Case presentation

An 88-year-old man with a Mobitz type II heart block was treated with a dual chamber pacemaker placement with active fixation leads. Post permanent pacemaker implantation chest x-ray showed appropriate lead position, and the electrocardiograph (EKG) showed a paced rhythm. The patient was discharged home and was able to continue with his daily activities without limitations until the day of presentation.

On the 12th day after the procedure, the patient developed chest pain. The pain was right-sided, non-radiating, and aching in character. This pain was described to be worse with inspiration. The patient denied palpitations, dizziness, nausea, syncope, or fatigue.

In the emergency department, his vitals were stable, and initial laboratory investigations revealed electrolytes to be within normal limits; cardiac troponin T was negative and the EKG showed intermittent lead capture. A chest X-ray revealed a left chest wall cardiac pacemaker device with the leads overlying the right atrium and right ventricle, and small bilateral pleural effusions. No pneumothorax was identified. Echocardiogram performed at bedside showed a small pericardial effusion, new compared to the study done on previous admission. There was no chamber compression, and the ejection fraction was 56%-60%, same as the previous echocardiogram. 

**Figure 1:**
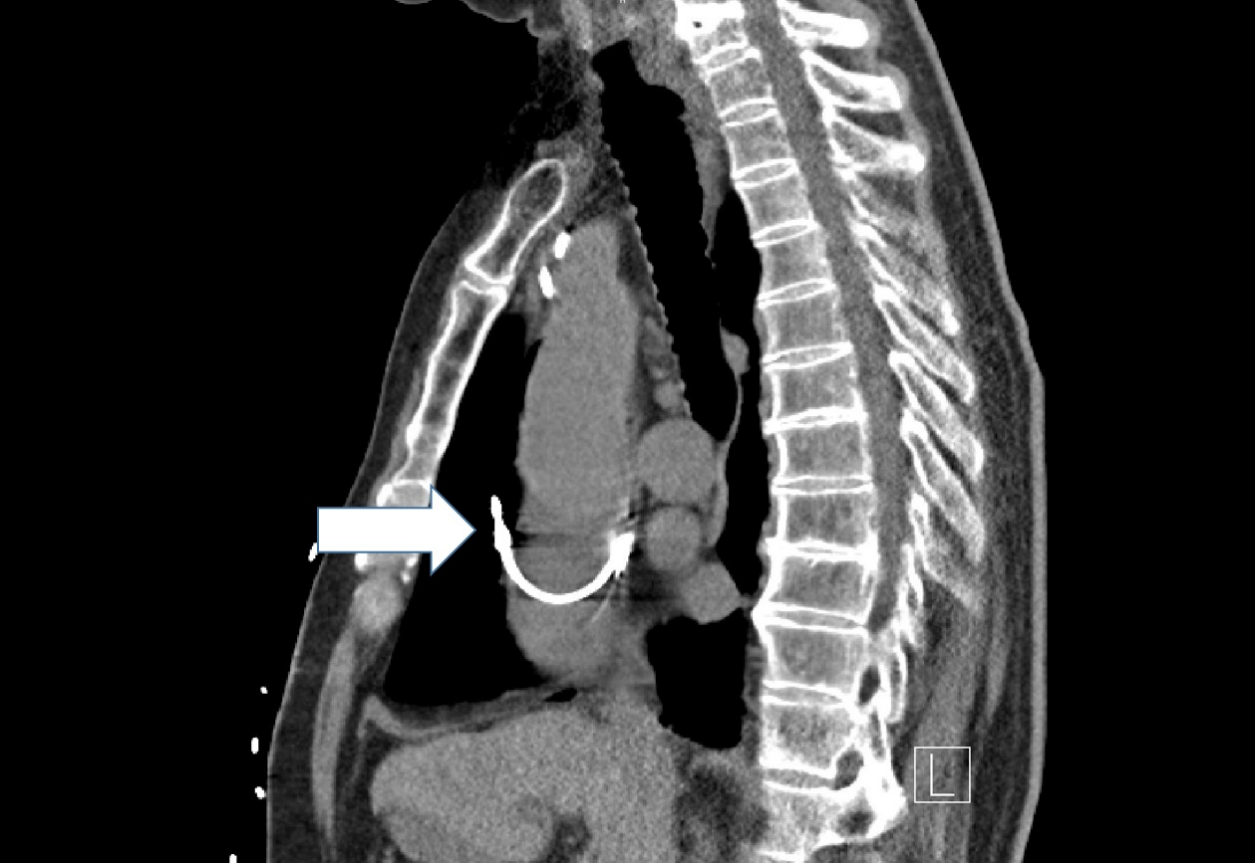
High-resolution computed tomography scan of the chest sagittal cut with the white arrow pointing at the tip of the atrial lead migrating into the right lung

**Figure 2:**
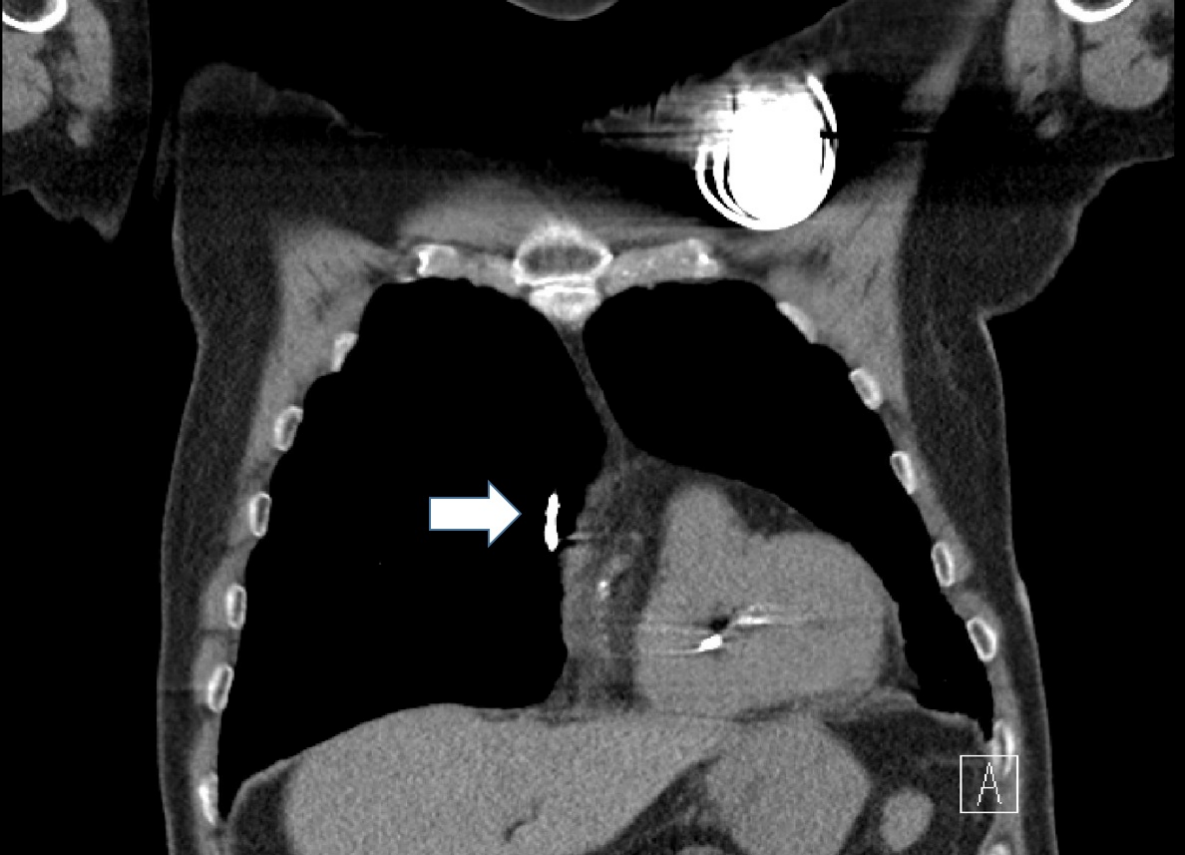
High-resolution computed tomography scan of the chest coronal cut with white arrow pointing at the tip of atrial lead migrating into the right lung

A high-resolution computed tomogram (HRCT) of the chest was performed, and it revealed migration of the right atrial lead into the right lung. The right atrial lead had penetrated through the right atrial appendage wall, and its tip was seen in the middle lobe of the right lung (Figures 1-2). Additionally, there was a small right pneumothorax, a small anterior pneumo-pericardium, a small pneumomediastinum along the ascending aorta, and a small right-sided pleural effusion. The patient underwent urgent right atrial lead revision and tolerated the procedure well. Repeat echocardiogram a day after the revision procedure showed a resolution of the pericardial effusion. The patient's chest pain resolved post procedure and he was discharged from the hospital. 

## Discussion

Pacemaker implantation is a fairly common procedure which carries low complication risks. Although rare, a serious complication of pacemaker implantation is myocardial perforation. Acute myocardial perforation occurs in about 1% of patients at the time of implantation [2]. The incidence of acute perforation has decreased over the years due to the development of modern pacing leads. Late perforation is defined as perforation which occurs after discharge from hospital after successful placement. It is a rare but serious complication [3]. Presentation can vary from mild discomfort to sudden hypotension and death. The speculated mechanism involves continuous pressure on to the myocardium by the lead. It is surprising how continuous pressure leads to penetration of the rigid pericardium and lung parenchyma along with the myocardium. Various risk factors associated with perforations have been identified, including female gender, old age, active fixation leads, use of systemic corticosteroids, prior temporary pacing, and the presence of cardiomyopathy [4]. Our patient fulfilled only two criteria, which were old age and active fixation leads.

**Figure 3:**
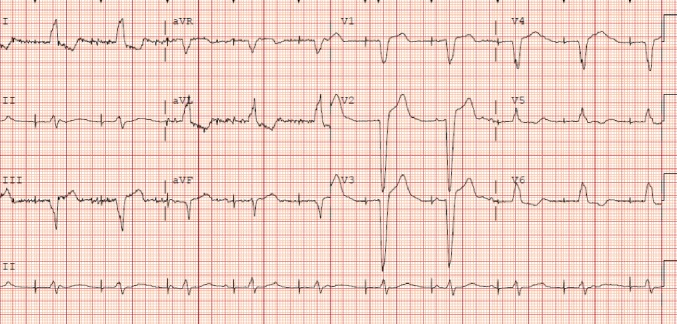
Electrocardiogram performed on presentation to the emergency room with chest pain showing intermittent beat capture

Diagnosis of lead perforation is aided by chest x-ray, electrocardiogram, echocardiogram, and the absence of capture on device interrogation along with clinical presentation. It is interesting to note that our patient had intermittent lead capture on EKG despite perforation of the above-mentioned structures and migration of the lead (Figure 3). A literature review of similar reports suggest that an HRCT scan is helpful in making a definitive diagnosis and should be obtained if there is suspicion of lead perforation. Apart from aiding in making the diagnosis, HRCT also helps in planning lead retrieval as it gives a good assessment of the orientation of vital structures around the displaced lead. Management varies from lead extraction and repositioning to thoracotomy with lead removal. The modality of intervention should be based on the timing of perforation, hemodynamic stability, comorbidities, and the presence of complicating factors such as pneumothorax and pericardial tamponade. We proceeded with lead extraction and repositioning as the patient did not display hemodynamic compromise and there was no evidence of pericardial tamponade or a large pneumothorax. 

## Conclusions

Late perforation by pacemaker leads is a rare phenomenon that can occur with minimal symptoms. This case highlights the importance of the HRCT scan in accurately diagnosing dislocation and planning the retrieval of the pacemaker lead after cardiac perforation. 
